# Interactive Effects of Temperature and Nutrient Conditions on Growth and Virulence Factor Expression of *Staphylococcus aureus* Under Model Food-Relevant Environments

**DOI:** 10.3390/foods15122062

**Published:** 2026-06-07

**Authors:** Zuo Hu, Hisaya K. Ono, Zhihao Zhu, Shouhei Hirose, Yukiko Hara-Kudo, Shaowen Li, Dong-Liang Hu

**Affiliations:** 1 Department of Zoonoses, Kitasato University School of Veterinary Medicine, Towada 034-8628, Japan; hu.zuo@st.kitasato-u.ac.jp (Z.H.); hisaono@vmas.kitasato-u.ac.jp (H.K.O.); zhuzhihao@webmail.hzau.edu.cn (Z.Z.); 2 College of Veterinary Medicine, Huazhong Agricultural University, Wuhan 430070, China; lishaowen@mail.hzau.edu.cn; 3 Division of Microbiology, National Institute of Health Sciences, Kawasaki 210-9501, Japan; sh-hirose@nihs.go.jp (S.H.); ykudo@hoshi.ac.jp (Y.H.-K.); 4 Laboratory of Microbiology, School of Pharmacy and Pharmaceutical Sciences, Hoshi University, Tokyo 142-8501, Japan

**Keywords:** *Staphylococcus aureus*, staphylococcal enterotoxins, meat and meat products, food safety

## Abstract

*Staphylococcus aureus* is a major cause of foodborne intoxication through the production of heat-stable enterotoxins (SEs) and is also an important opportunistic pathogen of humans and livestock. Meat and meat products are major vehicles for this pathogen because their protein-rich composition supports bacterial growth and toxin production. However, the combined effects of temperature and nutrient composition on *S. aureus* growth and virulence expression under food-relevant conditions remain unclear. In this study, we investigated the interactive effects of temperature and nutritional context on the growth and virulence-associated phenotypes under model food-relevant environments with the reference strain *S. aureus* FRI-S6. Bacterial growth, biofilm formation, staphylococcal enterotoxins A and B (SEA, SEB), and hemolytic activity were evaluated at 25 °C and 37 °C in brain heart infusion (BHI) medium supplemented with NaCl, glucose, or tryptone to simulate diverse food-relevant conditions. Growth was generally faster at 37 °C, whereas glucose-supplemented cultures at 25 °C reached higher cell densities during prolonged incubation. Biofilm formation increased at 37 °C in BHI and glucose conditions. SEA production was enhanced at 37 °C under NaCl and tryptone, but at 25 °C in glucose-rich conditions. In contrast, SEB production and hemolytic activity were consistently higher at 37 °C, particularly in the presence of tryptone and glucose. These findings demonstrate the strong interaction between temperature and nutrient composition in shaping *S. aureus* virulence in food environments and provide important insights for food safety risk assessment and highlight practical implications for controlling enterotoxin production in meat products and other foods during storage and processing.

## 1. Introduction

*Staphylococcus aureus* is one of the most common causative agents of foodborne intoxication worldwide due to its ability to contaminate food products and produce heat-stable staphylococcal enterotoxins (SEs) [1,2,3]. Beyond food safety, *S. aureus* is also a highly adaptable infectious pathogen that colonizes humans and animals and causes a broad range of diseases in clinical and veterinary settings [4,5,6]. Its capacity to persist across diverse ecological niches and to express virulence under contrasting environmental conditions underlies its importance as both a foodborne hazard and an opportunistic pathogen, particularly in animal-derived food systems such as meat and meat products, where contamination can occur along the production and processing chain [7,8,9].

Foodborne outbreaks associated with *S. aureus* are largely linked to its widespread presence in human and animal reservoirs, facilitating contamination during food production, processing, and handling. Foods implicated in staphylococcal food poisoning (SFP) include both protein-rich products (e.g., meat, eggs, milk, and dairy products) and carbohydrate-rich foods such as rice, rice-based products, and pastries [10,11,12]. Among these, meat and meat products are of particular concern due to their high protein content, handling intensity, and variable salt conditions, which may support bacterial growth and enterotoxin production under improper storage conditions. This concern is particularly relevant for processed meat products (e.g., cured, fermented, or ready-to-eat meats), where salt addition, processing steps, and extended storage may create selective conditions that influence *S. aureus* survival and modulate enterotoxin production. The outbreak of SFP remains a significant public health and food safety concern globally. In the United States, approximately 241,140 SFP cases occur annually, resulting in substantial economic losses [2,12]. In Europe, thousands of SFP outbreaks have been reported by the European Food Safety Authority (EFSA) [13,14,15]. In Asian countries, including Japan and China, SFP continues to occur throughout the year and remains an important food safety issue [16,17]. The predominant food vehicles associated with SFP differ markedly across regions. In Europe and North America, outbreaks are most frequently linked to animal-derived, protein-rich foods [13,14], whereas in many Asian countries, particularly Japan and China, carbohydrate-rich foods are more commonly implicated [16,17,18]. These differences are thought to reflect regional variation in food composition, processing and storage practices, and dietary habits. Notably, *S. aureus* can grow and produce SEs under environmental conditions commonly encountered during food storage and processing, presenting ongoing challenges for food safety management [19,20,21]. Understanding how food-relevant environmental factors influence the growth and virulence of *S. aureus* is essential for accurate risk assessment and effective control strategies.

Environmental signals, particularly temperature and nutrient availability, are key regulators of *S. aureus* physiology and virulence-associated traits, including enterotoxin production, hemolytic activity, and biofilm formation [22,23]. Temperature represents a major transition between food-associated environments at room temperature and host conditions at approximately 37 °C [24,25], while nutrient composition varies substantially among food matrices, including complex systems such as processed and fresh meat products with differing protein, salt, and carbohydrate profiles. Increasing evidence suggests that individual virulence factors, including specific enterotoxins, respond in a temperature- and nutrient-dependent manner rather than being uniformly upregulated under host-like conditions [22,26,27]. However, systematic comparative studies that integrate these environmental variables, especially those focusing on the classical staphylococcal enterotoxins A (SEA) and B (SEB)—the most important and commonly implicated toxins in staphylococcal food poisoning—remain limited. It is not well established whether SEA and SEB respond similarly or differently to the temperature × nutrient combination. A clearer understanding of the interaction effects of temperature and nutrient availability is therefore essential for improving food safety risk assessment and for developing targeted control strategies.

In the present study, we systematically investigated the combined effects of temperature and nutritional conditions on key pathogenic phenotypes of *S. aureus*. Using brain heart infusion (BHI) medium supplemented with NaCl, glucose, or tryptone as model environments representing the osmotic stress/salinity of cured products, carbohydrate-rich conditions, and nitrogen/protein-rich conditions, respectively, we compared growth kinetics, biofilm formation, SEA and SEB production, and hemolytic activity at 25 °C and 37 °C. These model conditions were selected to reflect key physicochemical characteristics relevant to diverse food systems. By dissecting the interaction between temperature and nutrient availability under controlled, food-relevant conditions, this study provides new insights into how environmental cues drive distinct virulence expression patterns in *S. aureus* under food-relevant conditions.

## 2. Materials and Methods

### 2.1. Bacterial Strain and Culture Conditions

*S. aureus* FRI-S6 (also referred to as FDA S6), originally isolated from a staphylococcal food poisoning outbreak, was used in this study. This strain harbors *sea* on the prophage ϕSa3mu and *seb*, *sek*, and *seq* on the pathogenicity island SaPI (vSa1) [28,29]. Owing to its well-characterized enterotoxin gene profile and reproducible toxin expression, FRI-S6 is widely used as a reference strain for studies of classical enterotoxin production and food-related virulence [21,22,30]. The strain was stored at −80 °C in tryptic soy broth (TSB) supplemented with 20% (*v*/*v*) glycerol. Prior to each experiment, frozen stocks were streaked onto tryptic soy agar (TSA) and incubated at 37 °C for 18–24 h to obtain isolated colonies. A single colony was inoculated into brain heart infusion broth supplemented with yeast extract (BHI) and incubated statically at 37 °C for 14 h to prepare overnight cultures, which served as the inoculum for subsequent experiments.

BHI was used as the base medium for all assays. Nutritional modifications were achieved by supplementation with sodium chloride (NaCl; 2.5%, 5%, or 10%), glucose (2%, 4%, or 8%), or tryptone (2.5%, 5%, or 10%), which were selected based on preliminary experiments conducted in our laboratory and were intended to represent low, moderate, and high nutritional or osmotic conditions relevant to different food environments. Prior to this study, preliminary experiments were conducted to evaluate the physicochemical characteristics of the tested media. Under the examined conditions, the pH values and water activity (aw) remained within a relatively narrow range, whereas NaCl supplementation produced substantially higher osmotic stress compared with glucose- or tryptone-supplemented media. Stock solutions were sterilized by filtration (0.22 µm) and diluted with autoclaved BHI to the desired concentrations. All media were confirmed to be sterile prior to use. Cultures were incubated statically at either 25 °C or 37 °C, representing food-associated ambient conditions and host physiological temperature, respectively.

### 2.2. Measurement of Bacterial Growth

Growth was monitored using a microplate-based optical density assay. Overnight cultures grown in BHI were diluted to approximately 6 × 10^5^ CFU/mL and further adjusted to a final inoculum of 6 × 10^4^ CFU/mL in each test medium. Aliquots (100 µL) were dispensed into 96-well plates in quadruplicate. Plates were incubated statically at 25 °C or 37 °C, and optical density at 550 nm (OD_550_) was measured at designated time points, using a microplate reader (Thermo Scientific, Life Technologies Holdings Pte. Ltd., Singapore).

### 2.3. Biofilm Formation Assay

Biofilm formation was assessed using the crystal violet staining method [22,31]. Briefly, diluted bacterial suspensions (final concentration: 6 × 10^4^ CFU/mL) were prepared in BHI and in each nutritionally modified medium as described for the bacterial growth assays. Aliquots of 100 µL were added to wells of sterile, flat-bottom 96-well polystyrene microplates in quadruplicate and incubated statically at 25 °C or 37 °C for 24 h. Following incubation, planktonic cells were gently removed, and wells were washed three times with sterile phosphate-buffered saline (PBS) to remove non-adherent cells. The remaining biofilms were fixed with 100 µL of methanol for 15 min, air-dried, and stained with 0.1% (*w*/*v*) crystal violet solution for 15 min at room temperature. Excess stain was removed by washing with distilled water, and the bound crystal violet was solubilized with 95% ethanol. Biofilm biomass was quantified by measuring absorbance at 590 nm (OD_590_) using a microplate reader (Thermo Scientific, Life Technologies Holdings Pte. Ltd., Singapore).

### 2.4. Detection of Staphylococcal Enterotoxins (SEA and SEB)

Production of staphylococcal enterotoxins was evaluated in culture supernatants obtained after growth under each nutritional condition. Bacterial cultures were centrifuged at 10,000× *g* for 10 min at 4 °C, and the supernatants were collected. The concentrations of staphylococcal enterotoxin A (SEA) and B (SEB) were quantified using a sandwich enzyme-linked immunosorbent assay (ELISA), as previously described, with minor modifications [22,32]. Briefly, 96-well microplates were coated overnight at 4 °C with anti-SEA or anti-SEB antibodies (20 µg/well) diluted in carbonate–bicarbonate buffer. Plates were washed three times with phosphate-buffered saline containing 0.05% Tween 20 (PBST) and blocked with 1% Block ace (BA) in PBS at 37 °C for 1 h. Culture supernatants and SEA or SEB standards (serial two-fold dilutions ranging from 0.78 to 100 ng/mL, prepared in 0.1% BA) were added to the wells (100 µL/well) in duplicate and incubated at 37 °C for 1 h. After washing with PBST, normal rabbit serum (1:100 dilution in 0.1% BA) was added to each well to inhibit nonspecific binding mediated by protein A. Plates were then washed and incubated with horseradish peroxidase (HRP)-conjugated anti-SEA or anti-SEB antibodies (1:1000 for anti-SEA or 1:500 for anti-SEB, diluted in 0.1% BA) at 37 °C for 1 h. Following washing, substrate solution containing ortho-phenylenediamine (OPD) and hydrogen peroxide in citrate–phosphate buffer was added (100 µL/well), and plates were incubated in the dark at room temperature for 20 min. The enzymatic reaction was stopped by the addition of 1 M sulfuric acid (100 µL/well), and absorbance was measured at 490 nm using a microplate reader (Thermo Scientific, Life Technologies Holdings Pte. Ltd., Singapore). Enterotoxin concentrations in samples were calculated based on standard curves generated using purified SEA and SEB standards. The toxin concentrations were evaluated primarily to compare the overall effects of temperature and nutritional conditions on enterotoxin accumulation.

### 2.5. Hemolytic Activity Assay

Hemolytic activity was evaluated using sheep red blood cells (RBCs) [33]. Briefly, RBCs (Japan Bioserum Co., Ltd., Fukuyama, Japan) were washed three times with PBS and resuspended to a concentration of 2% (*v*/*v*). Filter-sterilized culture supernatants obtained under each growth condition were mixed with equal volumes of the RBC suspension and incubated at 37 °C for 5 h with gentle mixing (final RBC concentration: 1%). After incubation, hemolytic activity was quantified by measuring absorbance at 540 nm using a microplate reader (Thermo Scientific, Life Technologies Holdings Pte. Ltd., Singapore). Complete hemolysis induced by 0.1% Triton X-100 was used as a positive control, while RBCs incubated with PBS served as a negative control. Hemolytic activity was expressed as a percentage of complete hemolysis. This assay does not distinguish between alpha-, beta-, delta-, or other hemolysins.

### 2.6. Statistical Analysis

All experiments were performed using three independent biological replicates conducted on different days under identical experimental conditions. Data obtained from these replicates were pooled and are presented as the mean ± standard deviation (SD), unless otherwise stated. Statistical analyses were performed using GraphPad Prism software (version 9.3.0; GraphPad Software, San Diego, CA, USA). Differences among multiple groups were evaluated using multifactorial analysis of variance (ANOVA). When applicable, post hoc multiple comparison analyses were performed to identify significant differences between individual groups. A value of *p* < 0.05 was considered statistically significant.

## 3. Results

### 3.1. Growth Kinetics of S. aureus Under Different Temperature and Nutritional Conditions

**Figure 1:**
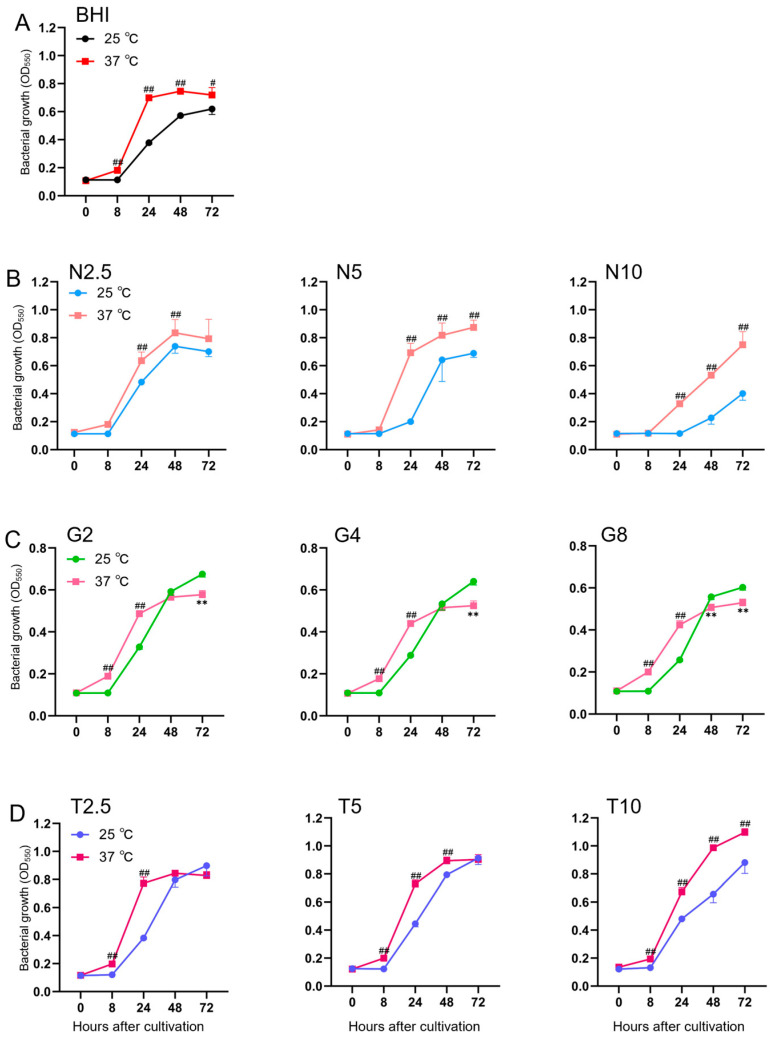
Temperature- and nutrient-dependent growth kinetics of *S. aureus.* Overnight cultures grown in BHI for 14 h were diluted to an initial concentration of 6.0 × 10^4^ CFU/mL and dispensed (100 μL per well) into 96-well flat-bottom microplates containing BHI alone (negative control), BHI inoculated with bacteria (positive control; (**A**)), or BHI supplemented with NaCl (2.5%, 5%, or 10%; (**B**)), glucose (2%, 4%, or 8%; (**C**)), or tryptone (2.5%, 5%, or 10%; (**D**)). Plates were incubated statically at 25 °C or 37 °C, and bacterial growth was monitored by measuring optical density at 550 nm (OD_550_) at 0, 8, 24, 48, and 72 h. Data are expressed as mean ± standard deviation (SD) for four replicate wells per condition at each time point. Statistical significance relative to the corresponding 25 °C group is denoted by ** *p* < 0.01 (lower than 25 °C), and ^#^ *p* < 0.05 and ^##^ *p* < 0.01 (higher than 25 °C).

Growth kinetics of *S. aureus* were compared across temperatures (25 °C and 37 °C) under control and nutrient-supplemented conditions (Figure 1). In BHI medium, bacterial growth increased steadily from 8 to 72 h, with consistently faster growth and higher cell densities observed at 37 °C than at 25 °C throughout the incubation period (Figure 1A). In NaCl-supplemented media, although no clear temperature-dependent differences were observed during the early phase of incubation (0–8 h), cultures incubated at 37 °C consistently exhibited higher growth than those at 25 °C after 8 h (Figure 1B). In glucose-supplemented media, temperature effects were time-dependent. Although growth at 37 °C was consistently higher during the early and mid-incubation phases (8–24 h), cultures incubated at 25 °C reached higher cell densities during the late phase (48–72 h) across all glucose concentrations tested (Figure 1C). This reversal of the temperature-associated growth advantage indicates that, while 37 °C generally supports more rapid early growth, prolonged incubation under glucose-rich conditions favors higher biomass accumulation at 25 °C. In tryptone-supplemented media, bacterial growth was consistently and significantly higher at 37 °C than at 25 °C across all tested concentrations throughout the entire incubation period (Figure 1D).

### 3.2. Biofilm Formation Under Different Temperature and Nutritional Environments

**Figure 2:**
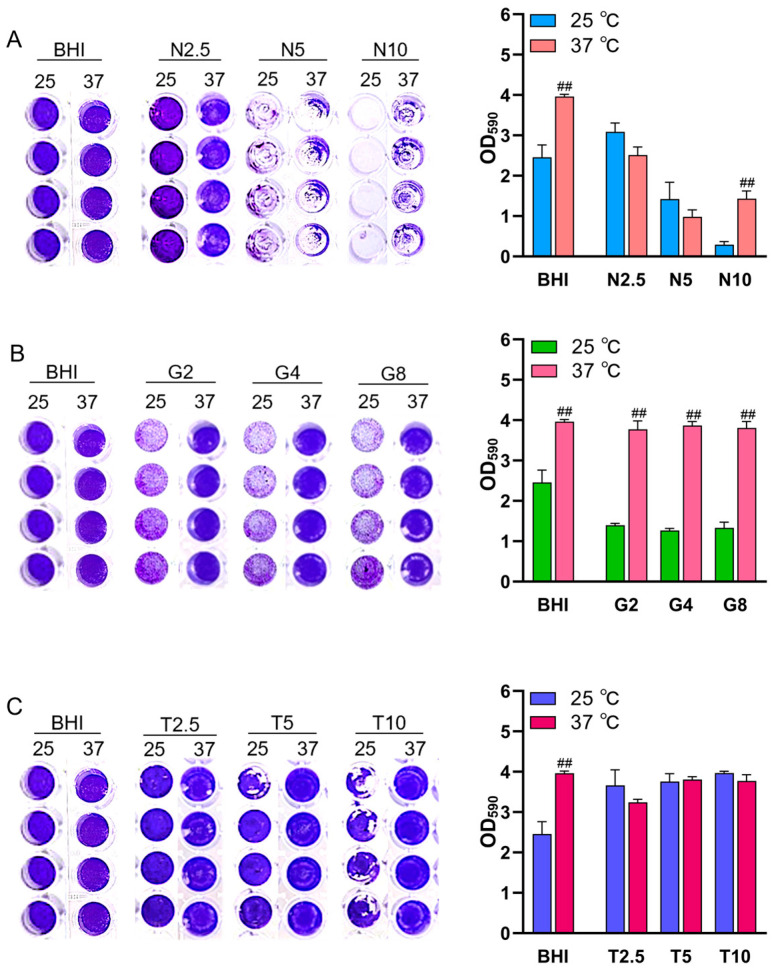
Temperature- and nutrient-dependent biofilm formation by *S. aureus*. Overnight cultures grown in BHI for 14 h were adjusted to 6.0 × 10^4^ CFU/mL and dispensed (100 μL per well) into 96-well flat-bottom microplates containing BHI alone (negative control), BHI inoculated with bacteria (positive control), or BHI supplemented with NaCl (2.5%, 5%, or 10%; (**A**)), glucose (2%, 4%, or 8%; (**B**)), or tryptone (2.5%, 5%, or 10%; (**C**)). Plates were incubated statically at 25 °C or 37 °C for 24 h. Biofilm formation was assessed using a crystal violet assay. Wells were stained with 0.1% (*w*/*v*) crystal violet, washed to remove unbound dye, air-dried, and photographed. Bound crystal violet was solubilized with 95% ethanol, and biofilm biomass was quantified by measuring optical density at 590 nm (OD_590_). Data are expressed as mean ± SD from four replicate wells per condition. Statistical significance relative to the corresponding 25 °C group is indicated. ^##^ *p* < 0.01 (higher than 25 °C).

Biofilm formation was quantified using the crystal violet assay. Under the BHI control condition, pronounced biofilm formation was observed at 24 h at both temperatures, with significantly higher biofilm levels at 37 °C than at 25 °C (Figure 2). These observations establish 37 °C as the more permissive temperature for biofilm development under baseline nutritional conditions. To evaluate how temperature interacts with specific nutritional factors, biofilm assays were performed in NaCl-, glucose-, or tryptone-supplemented media (Figure 2A–C). At 37 °C, 2.5%, 5% and 10% NaCl significantly reduced biofilm formation relative to BHI. At 25 °C, 5% and 10% NaCl also markedly inhibited biofilm formation, while the effect of 2.5% NaCl was minimal (Figure 2A). At 37 °C, supplementation with 2–8% glucose resulted in relatively high levels of biofilm formation, comparable to those observed in the BHI control at 37 °C. In contrast, at 25 °C, the same glucose concentrations significantly suppressed biofilm formation. Across all glucose-supplemented conditions, biofilm biomass was consistently higher at 37 °C than at 25 °C, indicating that temperature is the dominant determinant of biofilm formation in the presence of glucose (Figure 2B). Compared with the BHI control, supplementation with 2.5–10% tryptone at 25 °C significantly enhanced biofilm formation, indicating that under ambient-temperature conditions, protein-derived nutrients more effectively promote increased biofilm development. In contrast, at 37 °C, both the BHI control and the tryptone-supplemented groups exhibited strong biofilm formation, with no significant difference observed between the two conditions. Furthermore, within media supplemented with various concentrations of tryptone, no significant difference in biofilm biomass was observed between 25 °C and 37 °C (Figure 2C).

### 3.3. Regulation of SEA Production by Temperature and Nutritional Conditions

**Figure 3:**
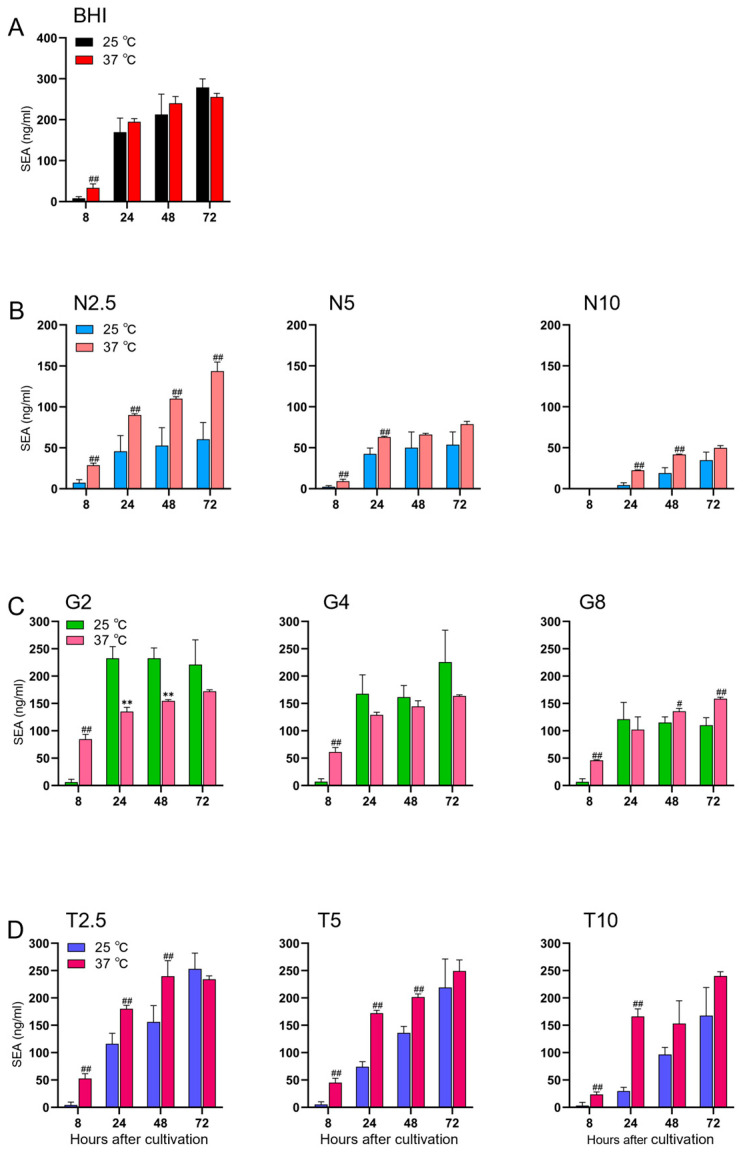
Temperature- and nutrient-dependent production of SEA by *S. aureus.* SEA concentrations in culture supernatants of *S. aureus* FRI-S6 grown in BHI alone (**A**) or BHI supplemented with NaCl (2.5%, 5%, or 10%; (**B**)), glucose (2%, 4%, or 8%; (**C**)), or tryptone (2.5%, 5%, or 10%; (**D**)) were determined by sandwich ELISA. Cultures were incubated statically at 25 °C or 37 °C, and supernatants were collected at 8, 24, 48, and 72 h. SEA levels were quantified using serial dilutions of recombinant SEA as standards. Absorbance was measured at 490 nm. Data are presented as mean ± SD from four independent wells per condition at each time point. Statistical significance is indicated by ** *p* < 0.01 (lower than 25 °C), and ^#^ *p* < 0.05 and ^##^ *p* < 0.01 (higher than 25 °C).

**Figure 4:**
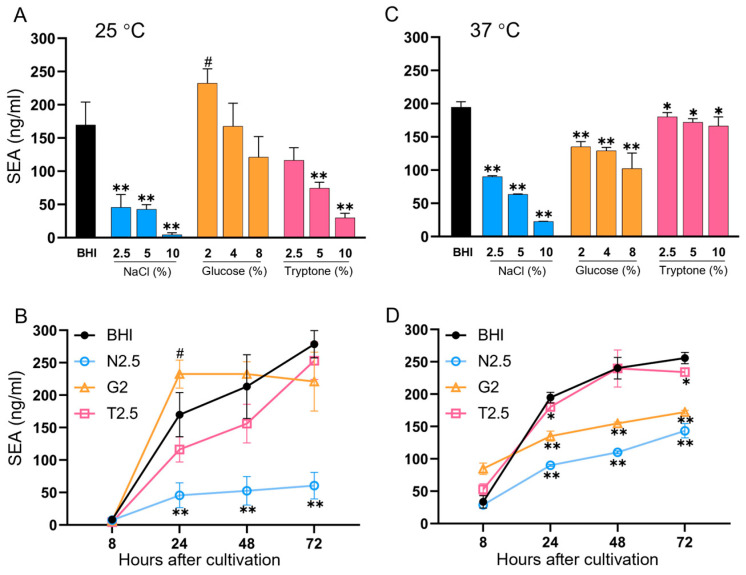
Comparative analysis of SEA production by *S. aureus* under different temperature and nutritional conditions. *S. aureus* FRI-S6 was cultured in BHI alone or supplemented with NaCl, glucose, or tryptone at 25 °C (**A**,**B**) or 37 °C (**C**,**D**). SEA concentrations in culture supernatants were quantified by ELISA. Panels (**A**,**C**) show SEA production after 24 h of incubation in BHI supplemented with different concentrations of each nutrient. Panels (**B**,**D**) show the time course of SEA production in BHI supplemented with NaCl (2.5%, N2.5), glucose (2%, G2), or tryptone (2.5%, T2.5) over the indicated incubation periods. Data are presented as means ± SD from four independent wells per condition. Statistical significance relative to the BHI control is indicated by * *p* < 0.05 and ** *p* < 0.01 (lower than BHI), and ^#^ *p* < 0.05.

SEA production by *S. aureus* was strongly influenced by both incubation temperature and nutrient composition, revealing a clear temperature × nutrient interaction (Figure 3). In BHI medium, SEA levels increased progressively from 8 to 72 h at both 37 °C and 25 °C, with no significant differences observed between the two temperatures (Figure 3A). In NaCl- and tryptone-supplemented media, SEA levels were consistently higher at 37 °C than at 25 °C across all concentrations tested. This temperature-associated increase was observed regardless of NaCl or tryptone concentration, indicating that host-associated temperature favors SEA production under these nutritional conditions (Figure 3B,D). In contrast, glucose supplementation produced an inverse temperature response. In glucose-containing media, SEA production was significantly higher at 25 °C than at 37 °C, with the difference becoming more pronounced at higher glucose concentrations. This pattern contrasted sharply with that observed in NaCl- and tryptone-supplemented environments (Figure 3C). When compared across nutrient conditions, cultures grown at 25 °C in glucose-supplemented medium exhibited the highest levels of SEA production, whereas SEA production in NaCl- and tryptone-supplemented media was lower than that observed in the BHI control (Figure 4A,B). At 37 °C, SEA production in NaCl-, glucose-, and tryptone-supplemented media was significantly lower than that in the BHI control (Figure 4C,D).

### 3.4. Regulation of SEB Production by Temperature and Nutritional Conditions

**Figure 5:**
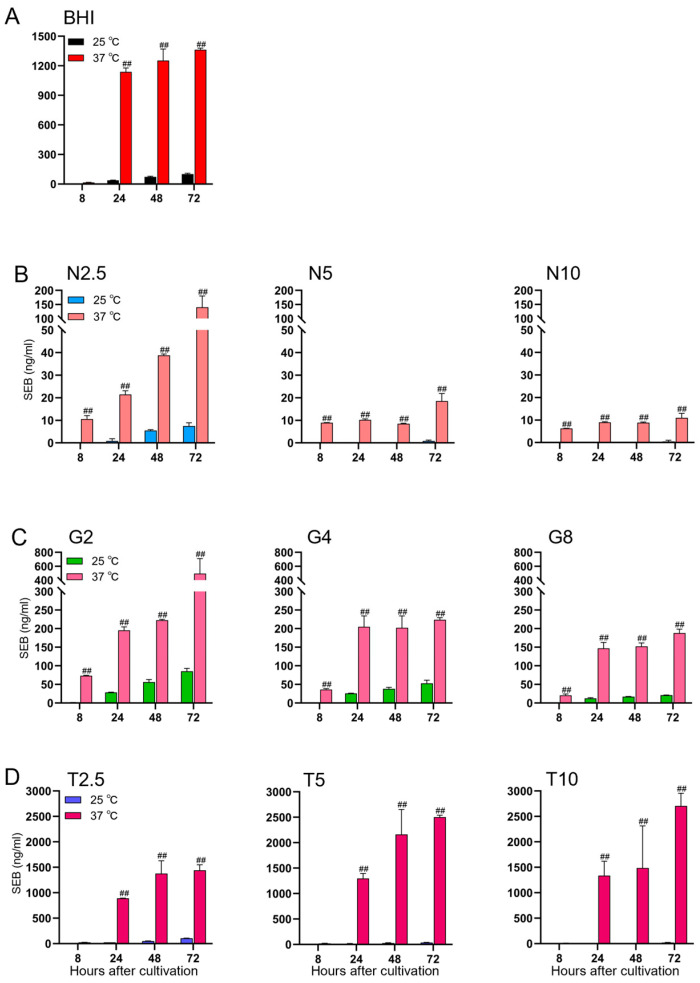
Temperature- and nutrient-dependent production of SEB by *S. aureus.* SEB concentrations in culture supernatants of S. aureus FRI-S6 grown in BHI alone (**A**) or BHI supplemented with NaCl (2.5%, 5%, or 10%; (**B**)), glucose (2%, 4%, or 8%; (**C**)), or tryptone (2.5%, 5%, or 10%; (**D**)) were quantified by sandwich ELISA. Cultures were incubated statically at 25 °C or 37 °C, and supernatants were collected at 8, 24, 48, and 72 h. SEB levels were calculated from standard curves generated using recombinant SEB. Absorbance was measured at 490 nm. Data are presented as mean ± SD from four independent wells per condition at each time point. Statistical significance is indicated by ^##^ *p* < 0.01 (higher than 25 °C).

**Figure 6:**
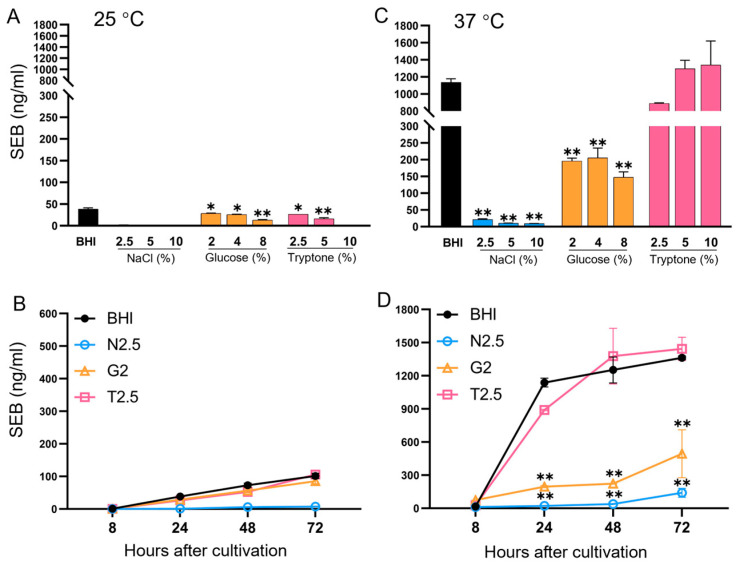
Comparative analysis of SEB production by *S. aureus* under different temperature and nutritional conditions. *S. aureus* FRI-S6 was cultured in BHI alone or supplemented with NaCl, glucose, or tryptone at 25 °C (**A**,**B**) or 37 °C (**C**,**D**). SEB concentrations in culture supernatants were quantified by ELISA. Panels (**A**,**C**) show SEB production after 24 h of incubation in BHI supplemented with different concentrations of each nutrient. Panels (**B**,**D**) show the time course of SEB production in BHI supplemented with NaCl (2.5%, N2.5), glucose (2%, G2), or tryptone (2.5%, T2.5) over the indicated incubation periods. Data are presented as means ± SD from four independent wells per condition. Statistical significance relative to the BHI control is indicated by * *p* < 0.05 and ** *p* < 0.01 (lower than BHI).

In contrast to SEA, SEB production exhibited a distinct temperature-dependent pattern that differed from that observed for SEA. Across all nutritional conditions tested, SEB levels were consistently higher at 37 °C than at 25 °C, indicating that physiological temperature strongly favors SEB expression (Figure 5). In NaCl-supplemented media, SEB production increased significantly at 37 °C relative to 25 °C, with this trend maintained across all salt concentrations (Figure 5B). In glucose-supplemented media, SEB production also remained significantly higher at 37 °C than at 25 °C, in contrast to the inverse temperature response observed for SEA under glucose-rich conditions (Figure 5C). Similarly, in tryptone-supplemented media, SEB levels were markedly elevated at 37 °C, suggesting that protein-rich environments at host-associated temperature promote SEB production (Figure 5D). These results indicate that SEB expression is predominantly regulated by temperature rather than by nutrient composition, with 37 °C consistently promoting significantly higher levels of production across diverse environmental conditions. This temperature-dominant regulatory profile distinguishes SEB from SEA and emphasizes toxin-specific differences in environmental responsiveness. When further compared across nutrient conditions, cultures incubated at 25 °C in NaCl- glucose- and tryptone-supplemented media exhibited lower levels of SEB production (Figure 6A,B). At 37 °C, SEB production in NaCl- and glucose-supplemented media was significantly lower than that in the BHI control; in contrast, SEB production in tryptone-supplemented medium was significantly higher than that in NaCl- or glucose-supplemented media and was comparable to that observed in the BHI control (Figure 6C,D).

### 3.5. Effects of Temperature and Nutritional Conditions on Hemolytic Activity

**Figure 7:**
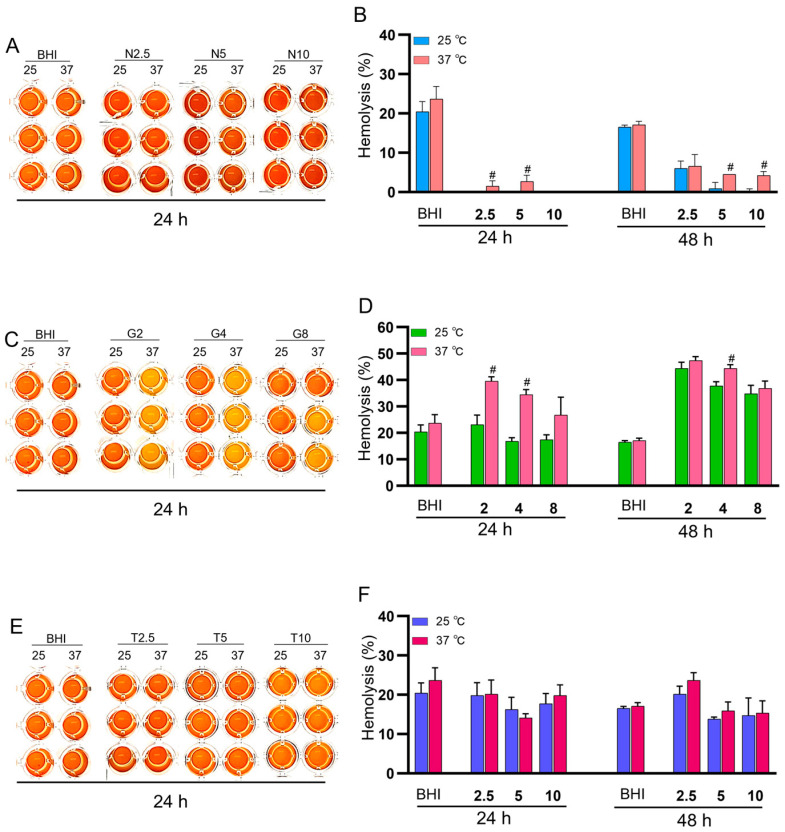
Hemolytic activity of *S. aureus* under different temperature and nutritional conditions. Hemolytic activity in culture supernatants of *S. aureus* FRI-S6 grown in BHI alone or BHI supplemented with NaCl (2.5%, 5%, or 10%; (**A**,**B**)), glucose (2%, 4%, or 8%; (**C**,**D**)), or tryptone (2.5%, 5%, or 10%; (**E**,**F**)) was assessed using sheep red blood cells (RBCs). Cultures were incubated at 25 °C or 37 °C, and cell-free supernatants were collected at 24 and 48 h. Washed RBCs (1%, *v*/*v* in PBS) were incubated with an equal volume of supernatant at 37 °C for 1 h. Hemoglobin release was quantified by measuring absorbance at 540 nm. Complete hemolysis induced by 0.1% Triton X-100 and PBS-treated RBCs were used as positive and negative controls, respectively. Hemolytic activity is expressed as a percentage of complete hemolysis. Data are presented as mean ± SD from three to six independent wells per condition. Statistical significance is indicated by ^#^ *p* < 0.05.

In BHI medium, substantial hemolytic activity was detected, with no significant difference between 25 °C and 37 °C (Figure 7). In NaCl-supplemented media, hemolytic activity was strongly suppressed to near background levels at 25 °C, indicating an inhibitory effect of osmotic stress. Nevertheless, hemolytic activity at 37 °C remained significantly higher than that at 25 °C (Figure 7A,B). In glucose-supplemented media, hemolytic activity after 24 h of cultivation was significantly higher at 37 °C than at 25 °C, and this difference increased with prolonged incubation and higher glucose concentrations (Figure 7C,D). Moreover, hemolytic activity after 48 h at both temperatures exceeded that observed in BHI, indicating that glucose availability enhances hemolytic activity in *S. aureus*. In tryptone-supplemented media, hemolytic activity was comparable to that in BHI and showed no significant temperature-dependent differences (Figure 7E,F).

## 4. Discussion

The present study provides a systematic comparison of how temperature and nutritional composition jointly regulate growth and key virulence-associated phenotypes in *S. aureus*, bridging conditions relevant to food environments and host infection. By integrating growth dynamics, biofilm formation, enterotoxin production, and hemolytic activity within a unified experimental framework, our findings reveal that virulence expression in *S. aureus* is not governed by temperature alone but is highly contingent on nutrient context, with distinct regulatory patterns emerging for individual toxins and pathogenic traits. This is particularly relevant for complex food matrices such as meat and meat products, where variable protein content, salt levels, and processing conditions may shape pathogen behavior in heterogeneous ways. A central outcome of this work is the identification of toxin-specific environmental responsiveness. While physiological temperature (37 °C) broadly favored bacterial growth, biofilm accumulation, SEB production, and hemolytic activity—features typically associated with host adaptation and infection—SEA production exhibited a markedly different profile, showing strong nutrient dependence and enhanced expression under glucose-rich conditions at ambient temperature. This divergence highlights that classical enterotoxins SEA and SEB are differentially wired to environmental cues, rather than being uniformly upregulated under host-like conditions. Such toxin-specific regulation provides a plausible mechanistic explanation for why certain enterotoxins, particularly SEA, are disproportionately implicated in foodborne intoxication events, where nutrient composition and suboptimal temperatures prevail [27,34].

Importantly, the observed temperature–nutrient interactions highlight the ecological versatility of *S. aureus* and its capacity to fine-tune pathogenic potential across disparate environments. In food-associated settings, glucose-driven growth advantages and SEA production at lower temperatures may elevate intoxication risk even in the absence of rapid proliferation, whereas in host-associated environments, temperature-dominant regulation of SEB and hemolytic activity likely contributes to invasive potential and tissue damage [22,35,36]. In the context of meat and meat products, such interactions may be further influenced by processing factors such as curing, salting, and storage conditions, which can create selective environments that modulate toxin expression. Together, these findings emphasize that food safety risk assessments and pathogenicity models should move beyond single-factor evaluations and instead account for the combined influence of thermal and nutritional parameters on *S. aureus* virulence expression. The differential responses of virulence-associated traits to temperature and nutrient composition observed in this study are consistent with the multilayered regulatory architecture governing *S. aureus* pathogenicity [37,38]. Environmental cues such as temperature, carbon source availability, and osmotic stress are known to converge on global regulatory systems, including the accessory gene regulator (*agr*), catabolite control protein A (*CcpA*), and stress-responsive sigma factors, which together modulate toxin expression, biofilm development, and extracellular enzyme production [39,40,41]. Our findings suggest that these regulatory networks integrate thermal and nutritional signals in a factor-specific manner, resulting in distinct expression profiles for individual virulence determinants.

The temperature-dominant regulation of SEB production and hemolytic activity, both of which were consistently enhanced at 37 °C across nutritional conditions, is indicative of virulence programs preferentially optimized for host-associated environments [38,42]. Previous studies have shown that *agr* activity is maximal at physiological temperature and promotes the expression of secreted toxins and hemolysins, while repressing surface-associated traits [5,27,36,43]. The parallel temperature-dependent upregulation of SEB and hemolytic activity observed here is consistent with *agr*-mediated regulation, although direct measurement of regulatory activity will be required to confirm this linkage. In contrast, SEA production displayed a pronounced dependence on nutrient composition, particularly under glucose-rich conditions at 25 °C, highlighting a regulatory logic distinct from that of SEB. SEA expression is known to be strongly influenced by prophage-encoded regulatory elements and is less tightly coupled to *agr* than other exotoxins [43,44]. Moreover, glucose-mediated carbon catabolite repression, mainly governed by *CcpA*, has been reported to differentially affect enterotoxin genes [45,46]. The enhanced SEA production observed under glucose-rich, suboptimal temperature conditions may therefore reflect the integration of metabolic signaling and phage-associated regulation, favoring enterotoxin expression in food-relevant environments rather than during invasive infection [22,45].

From a food safety perspective, the present findings emphasize that the risk posed by *S. aureus* in food systems cannot be inferred solely from bacterial growth or from extrapolation of host-associated virulence profiles. Under food-relevant conditions characterized by ambient temperature and specific nutrient compositions, *S. aureus* exhibited virulence expression patterns that diverged markedly from those observed at physiological temperature. In particular, the enhanced growth and elevated SEA production observed in glucose-supplemented media at 25 °C highlight a scenario in which classical enterotoxin production may be favored despite suboptimal growth temperatures. Given the heat stability of staphylococcal enterotoxins, such conditions may permit significant toxin accumulation even when bacterial proliferation appears limited, thereby increasing the risk of foodborne intoxication [34,47]. These findings suggest potential risk scenarios that should be validated in real food matrices, which frequently support *S. aureus* survival during storage and processing at non-refrigerated or mildly abusive temperatures. In addition, certain processed meat products—particularly ready-to-eat or minimally heated items—may also be at risk when post-processing contamination occurs and temperature control is inadequate, allowing toxin accumulation without obvious spoilage. The observation that SEA and SEB respond differently to identical environmental cues further emphasizes that toxin-specific regulatory behaviors should be considered in food safety risk assessments, rather than assuming uniform enterotoxin expression. This distinction is particularly relevant because SEA is the most frequently implicated enterotoxin in staphylococcal food poisoning outbreaks worldwide [6,19,21].

Moreover, the nutrient- and temperature-dependent modulation of biofilm formation observed in this study has important implications for the persistence of *S. aureus* in food-processing environments. Elevated temperature generally promoted biofilm accumulation, whereas nutrient effects were highly temperature dependent: glucose suppressed biofilm formation at 25 °C, while tryptone significantly enhancing it. These responses are consistent with the antagonistic relationship between *agr* activation and biofilm development, as well as with nutrient-mediated regulation of surface-associated proteins [48,49,50,51]. These findings support a model in which *S. aureus* flexibly reallocates resources between growth, persistence, and virulence in response to environmental constraints rather than uniformly maximizing pathogenic potential [52,53]. Such biofilm-associated persistence is particularly problematic in meat-processing environments, where equipment surfaces and cold-chain interruptions can facilitate repeated contamination events. Enhanced biofilm formation under specific nutrient conditions may promote long-term colonization of processing surfaces and increase the risk of recurrent contamination, whereas conditions that suppress biofilm formation but favor enterotoxin production may result in transient yet highly hazardous contamination events. Together, these results highlight the importance of incorporating temperature and nutrient composition into hazard analysis and critical control point (HACCP)-based risk assessment and predictive models of staphylococcal food poisoning.

Despite the valuable insights provided by this study, several limitations should be considered. Experiments were conducted using a single reference strain, which may not fully reflect the strain-level diversity of enterotoxigenic isolates from different food matrices or geographical origins. In addition, this study primarily focused on phenotypic outcomes without directly addressing the underlying regulatory mechanisms governing temperature- and nutrient-dependent responses. Future studies integrating transcriptomic analyses and regulatory mutants will be required to clarify the molecular basis of the observed toxin-specific regulation. Moreover, simplified laboratory media were used to model food- and host-associated environments, whereas real food systems involve greater complexity, including heterogeneous nutrient availability, competing microbiota, and dynamic physicochemical conditions. Future work incorporating real meat and meat product systems would further enhance the applicability of these findings to industrial food safety management. More studies incorporating diverse strains and real food systems will help further refine predictive models, strengthen risk assessment frameworks, and improve strategies for the prevention and control of staphylococcal food poisoning in food environments.

## 5. Conclusions

This study demonstrates that the growth and virulence expression of *S. aureus* are shaped by a strong interaction between temperature and nutritional conditions under laboratory model environments, resulting in distinct pathogenic profiles under food-associated versus host-associated conditions. While incubation at 37 °C generally promoted rapid growth, biofilm formation, SEB production, and hemolytic activity, ambient temperature (25 °C) in glucose-rich environments favored sustained growth and enhanced SEA production. These toxin-specific and environment-dependent responses highlight that virulence regulation in *S. aureus* is not uniformly optimized at physiological temperature but is instead finely tuned to specific combinations of environmental cues. Importantly, the observation that glucose at ambient temperature can support prolonged growth and elevated SEA production reinforces a critical risk scenario for staphylococcal food poisoning. Such conditions may be encountered not only in carbohydrate-rich foods but also in certain processed meat products, particularly those subject to post-processing contamination and temperature abuse during storage or handling. Together, these findings provide mechanistic insight into the ecological adaptability of *S. aureus* and emphasize the need to consider both temperature and nutrient composition in food safety risk assessment and control strategies. These insights are especially relevant for improving risk management and toxin control in diverse food systems, where complex nutrient composition and processing conditions can influence pathogen behavior.

## Data Availability

The original data supporting this study have been deposited in a public repository and are available in Zenodo at https://doi.org/10.5281/zenodo.19364618.
